# Complex Form Variant of Dysembryoplastic Neuroepithelial Tumor of the Cerebellum

**DOI:** 10.1155/2012/718651

**Published:** 2012-09-05

**Authors:** Jesús Vaquero, Cristobal Saldaña, Santiago Coca, Mercedes Zurita

**Affiliations:** ^1^Neurosurgical Service, Hospital Puerta de Hierro-Majadahonda, 28222 Madrid, Spain; ^2^Neuroscience Research Unit, Hospital Puerta de Hierro-Majadahonda, 28222 Madrid, Spain; ^3^Department of Pathology, Hospital Gómez Ulla, 28047 Madrid, Spain

## Abstract

Dysembryoplastic neuroepithelial tumor (DNT) is a benign neoplasm with typical supratentorial location, but the possibility of these rare tumors can also be located in the posterior fossa must be taken into account. We report a 21-year-old woman that suffered gait instability, headache, and diplopia. On CT-scan, an intraparenchymatous cerebellar tumor was disclosed. It was isodense, showing light enhancement after contrast administration. On MRI (T1-weighted image) the tumor was isointense, showing inhomogeneous hyperintensity after-gadolinium administration. On T2-weighted MRI, the tumor was inhomogenously hyperintense. At surgery, a solid and hypervascularized tumor was completely removed. Two years after surgery, the patient is symptom-free. Pathological study showed coexistence of areas of pilocytic astrocytoma with areas in which small rounded oligodendrocyte-like cells (OLC), with strong synaptophysin expression were identified. These neurocytic areas showed an eosinophilic matrix forming microcysts, and cells with aspect of “floating neurons” were occasionally identified. A complex form variant of DNT was diagnosed. Our case suggests that in presence of a cerebellar tumor with features of pilocytic astrocytoma, the possibility of a complex form variant of DNT should be considered.

## 1. Introduction

Dysembryoplastic neuroepithelial tumor (DNT) is a rare and benign neoplasm with typical supratentorial location [1, 2]. Nevertheless, in the first description of DNT by Daumas-Duport et al. in 1988, these authors briefly described two cerebellar tumors in children, with features of microcystic cerebellar astrocytoma but with a striking similarity to DNT, due to the presence of cortical dysplasia with oligodendrocytic and neuronal components [2]. In 1994, a cerebellar tumor diagnosed as DNT was reported [3] and since then, at least another five cases of DNT affecting the cerebellum have been described [4–9]. In these cases, the hypothesis that they represent neurocytic neoplasms arising from the external granular layer of the cerebellum has been suggested [2]. We describe here a rare case of cerebellar glioneuronal tumor that can be included in the complex form variant of DNT, according to the current WHO classification [1].

## 2. Case Presentation

**Figure 1:**
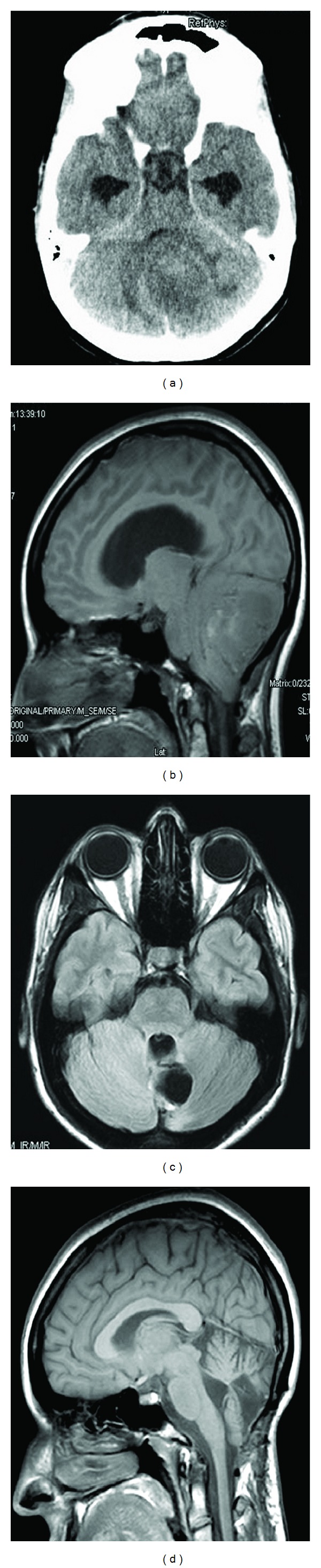
(a) CT-scan, after contrast administration, showing the cerebellar tumor, with peritumoral edema and hydrocephalus. (b) T1-weighted MRI, after gadolinium administration, showing the hypointense cerebellar tumor, with areas of hyperintensity. (c) and (d) T1-weighted MRI, after contrast administration, two years after surgery. Absence of residual tumor or tumor recidive can be seen.

Our patient was a 21-year-old woman, suffering two weeks of gait instability, headache, and diplopia. CT-scan showed an isodense intraparenchymatous cerebellar tumor with light enhancement after contrast administration (Figure 1(a)). T1-weighted MRI showed an isointense tumor, with inhomogeneously hyperintensity after-gadolinium administration (Figure 1(b)). The lesion was inhomogeneously hyperintense on T2-weighted MRI. Peritumoral edema and hydrocephalus were also observed.

After posterior fossa surgery, a solid and hypervascularized tumor, with necrotic areas, was completely removed. Two years after surgery, the patient is symptom-free and control MRI shows absence of recurrence or residual tumor (Figures 1(c) and 1(d)). 

Pathological study of the resected tumor showed a nonhomogenous tissue with areas of ischemic necrosis and a great number of hyalinized and glomeruloid vessels. In the hamartomatous-like vessels, endothelial cells showed frequent Ki-67-positivity.

**Figure 2:**
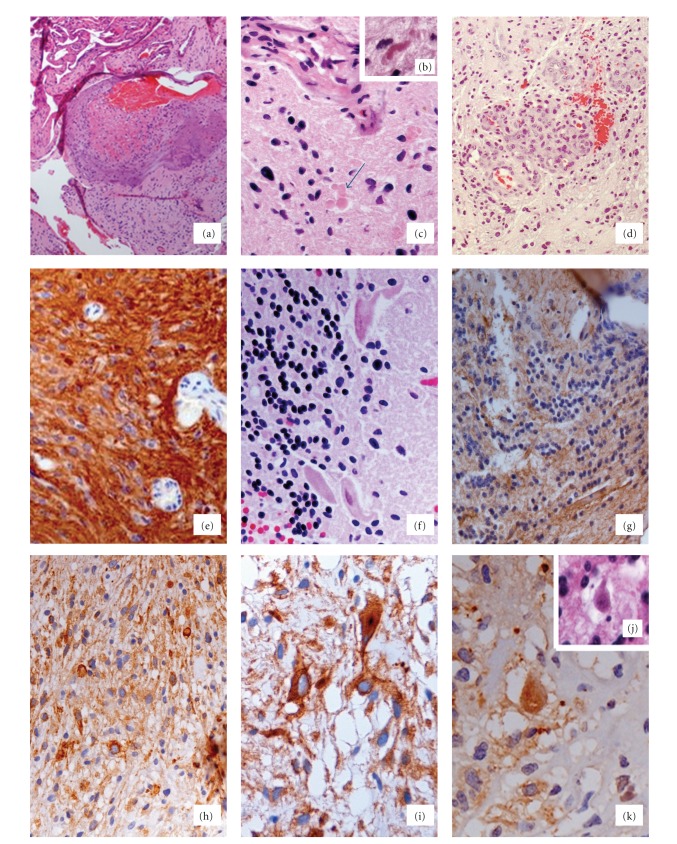
Microscopical findings of the resected tumor. (a) Hamartomatous-hyalinized vessels among the tumor cells can be seen (h and e, ×40). (b) Rosenthal fibers (h and e, ×400). (c) Hyalinized vessels and eosinophilic granular bodies (arrow) (h and e, ×200). (d) Vessels with glomeruloid aspect among tumor cells (h and e, ×100). (e) Strong GFAP expression in the areas with aspect of pilocytic astrocytoma (GFAP, ×200). (f) Area of removed tissue showing normal cerebellar cortex. Some Purkinje cells with abnormal position, and granular cells with normal aspect can be seen (h and e, ×200). (g) Tumor area showing small oligodendroglial-like cells with absence of GFAP positivity (GFAP, ×200). (h) Tumor area with small oligodendroglial-like cells and strong positivity to synaptophysin (×100). (i) Cells with larger size and strong synaptophysin expression can be seen. (×200). (j) and (k) Floating neurons can occasionally observed within microcysts, h and e (j) and synaptophysin expression (k) ×400.

Areas of pilocytic astrocytoma, with Rosenthal fibers and eosinophilic granular bodies were occasionally identified. These areas showed strong gliofibrillary acidic protein (GFAP)-positivity. In other areas, cerebellar cortex with abnormal positioning of Purkinje cells was identified, but unquestionable images of cortical dysplasia were not seen. In other areas, small rounded oligodendrocyte-like cells (OLC), showing strong synaptophysin expression were identified. These cells were dispersed in an eosinophilic matrix forming microcysts. Occasionally, larger cells were observed in the alveolar matrix, some of them with aspect of “floating neurons” and showing strong synaptophysin expression (Figure 2). With these data, a diagnosis of “complex DNT” was established.

## 3. Discussion

DNT is a glioneuronal tumor whose main characteristic is the presence of OLC arranged around bundled axons and capillaries. The neuronal characterization of the small rounded cells and the presence of neurons floating in a myxoid matrix are the features useful in differentiating DNT from oligodendroglioma, which is important because DNT has an excellent prognosis following surgical removal.

Our present case shows a cerebellar tumor with areas of pilocytic astrocytoma, and areas with small synapthophysin-positive cells suggesting a neurocytic component. Although the presence of synaptophysin-positive cells of variable size suggests a diagnosis of ganglioneurocytoma, the identification of “floating neurons” allowed us to a diagnosis of DNT. At present, the coexistence of features of pilocytic astrocytoma and DNT has been accepted in the so-called “complex form” of DNT [1]. 

It is obvious that our case adds to the rare descriptions of cerebellar DNT and supports the variability of the so-called glioneuronal tumors [10]. Due to the good outcome of both DNT and cerebellar pilocytic astrocytoma, we may suspect that cases previously diagnosed as pilocytic astrocytoma are really DNT. In any case, our present observation suggests that in presence of a cerebellar tumor with features of pilocytic astrocytoma, the possibility of a complex variant of DNT must be considered.
